# Women diagnosed with HIV and unknown HIV status perceived susceptibility to cervical cancer and perceived benefits of cervical cancer screening in Ghana: a cross-sectional study

**DOI:** 10.1186/s12905-021-01509-9

**Published:** 2021-10-17

**Authors:** Nancy Innocentia Ebu Enyan, Selorm Akaba, Sarah Ama Amoo

**Affiliations:** 1 Department of Adult Health, School of Nursing and Midwifery, University of Cape Coast, Cape Coast, Ghana; 2 Department of Agricultural Economics and Extension, School of Agriculture, University of Cape Coast, Cape Coast, Ghana; 3 Cape Coast Teaching Hospital, Cape Coast, Ghana

## Abstract

**Background:**

Cervical cancer is an issue of global health concern, and it seems to be the next epidemic in Sub-Saharan Africa after Human Immunodeficiency Virus (HIV). This study compared the perceptions of susceptibility to cervical cancer and benefits of cervical cancer screening among women diagnosed and those with unknown HIV status and determined the association between socio-demographic factors and HIV status.

**Methods:**

A cross-sectional study was conducted with 600 women diagnosed with HIV and 600 women with unknown HIV status in the Central Region of Ghana. Convenience sampling was used and a structured interview schedule was the main data collection instrument. Data were analysed using frequencies, percentages, chi-square test and independent samples t-test.

**Results:**

A high proportion of women diagnosed with HIV 94.8% (n = 569) and those with unknown HIV status 93.5% (n = 561) agreed that “screening can find cervical changes”. Also, 58.0% (n = 348) of women diagnosed with HIV agreed that they have been in polygamous relationships so they may get cervical cancer. There was a statistically significant association between marital status (X^2^ = 167.071, *p* = 0.001), religion (X^2^ = 57.720, *p* = 0.001), level of education (X^2^ = 118.997, *p* = 0.001), employment status (X^2^ = 782.646, *p* = 0.001) and HIV status. A comparison of the mean difference for women diagnosed and those with unknown HIV status in relation to perceived benefits of cervical cancer screening showed a statistically significant difference (t = 7.418, df = 1198, *p* = 0.001). Nonetheless, there was no statistically significant difference in the means for women diagnosed and those with unknown HIV status regarding perceived susceptibility to cervical cancer (t = 0.935, df = 1198, *p* = 0.351).

**Conclusions:**

Women with HIV perceived higher benefits of cervical cancer screening. Perception of susceptibility to cervical cancer by women with and those without HIV need to be addressed in efforts to improve their health. Furthermore, interventions for women with HIV should consider some important sociodemographic factors.

## Background

Women with the Human Immunodeficiency Virus (HIV) have higher risk of cancer of the cervix because of their increased tendency of acquiring Human Papillomavirus (HPV) infection and faster progression of the disease to cancer compared to those with negative HIV status [1]. It is estimated that 85% of women living with cervical cancer are in the Sub-Saharan Africa (SSA), a region with a high burden of HIV. Efforts to achieve the World Health Organization [WHO]’s target of 70% cervical cancer screening coverage and 90% access to treatment for cervical precancer abnormalities by 2030 require interventions to improve screening uptake [1]. Interventions to increase HPV vaccination, screening and treatment will reduce the incidence of cancer of the cervix by 40% and its related mortality by 5 million in 2050 [1].

In Ghana, cervical cancer is a major cause of death among women, especially those within age group 15–44 [2]. Women with HIV have higher chances of acquiring cervical cancer due to the immunosuppression that results from HIV infection [3]. The Joint United Nations Programme on HIV/AIDS (UNAIDS) report suggests that women form 52% of all people living with HIV (PLHIV) in low-to-middle-income economies, but SSA has the highest proportion (57%) of women living with HIV (WLHIV)/acquired immunodeficiency syndrome (AIDS) [4].

It is estimated that 320,000 adults are living with HIV/AIDS in Ghana, and women constitutes 65.6% of this population [5]. HIV/AIDS prevalence among adults 15–49 years in the country is estimated to be 1.7% [5]. The HIV/AIDS epidemic directly impact on the health and wellbeing of women. WLHIV have an increased risk of developing cervical cancer, requiring an urgent need for an expanded access to HIV testing, comprehensive treatment, and availability of sexual and reproductive health services [6]. The much higher incidence of cancer of the cervix among HIV-infected women is related to the high presence of infection with cancer-causing types of HPV [7, 8]. Several studies have affirmed that women with HIV have greater risk for HPV infection and malignancies associated with HPV including cervical cancer [3, 9].

Risk perception is a significant factor that can enable people to adopt health promoting behaviours as postulated by the Health Belief Model (HBM) and Protection Motivation Theory [10, 11]. Perceived susceptibility or perception of risk can be defined as an individual’s subjective perception of the risk of developing a health condition [12]. Perceived susceptibility, as a construct, has been linked with many health behaviours and preventive health actions such as cervical cancer screening [13]. Perceptions of risk of cervical cancer can be associated with great pain, anxiety, and worry about cancer [14]. Several studies have examined the perceived risk of cervical cancer and the intent for screening [9, 15]. A previous study reported that HIV-positive women on treatment and have access to cervical cancer screening services may not perceive themselves as susceptible to cervical cancer [16].

Furthermore, women may engage in healthy behaviours if they deem it to be beneficial to their health. Cervical cancer screening is critical in decreasing incidence of cervical cancer and the mortality associated with it [17]. The screening test helps in the detection of abnormal cervical lesions for early and effective management [18, 19]. In an earlier study conducted in South Africa, perception of benefits of screening influenced screening intention [20]. However, Ibekwe et al. reported that perceived benefits of engaging in cervical cancer screening may not be a strong predictor of cervical cancer screening [18]. In Ghana, although several studies have investigated the health behaviours of women [15, 21], little is known about women diagnosed with HIV and those with unknown HIV status perceptions of susceptibility to cervical cancer and benefits of cervical cancer screening. This study sought to investigate; (1) the association between perceived susceptibility to cervical cancer and HIV status, (2) the association between perceived benefits of cervical cancer screening and HIV status, (3) association between sociodemographic factors and HIV status and 4) the difference in perceived susceptibility to cervical cancer and perceived benefits of cervical cancer screening in relation to HIV status.

## Methods

### Research design

This study was conceptualized within the HBM as it has been used widely in explaining health behaviours [10]. The model comprised concepts including sociodemographic factors and perceptions of susceptibility, seriousness, benefits, barriers and cues. An important concept in the HBM is the likelihood of action. This study adopted sociodemographic factors and perceptions of susceptibility and benefits from the model. A descriptive cross-sectional survey was conducted involving women diagnosed with HIV and those with unknown HIV status aged 20–65 years in the Central Region of Ghana.

### Population and setting

**Fig. 1:**
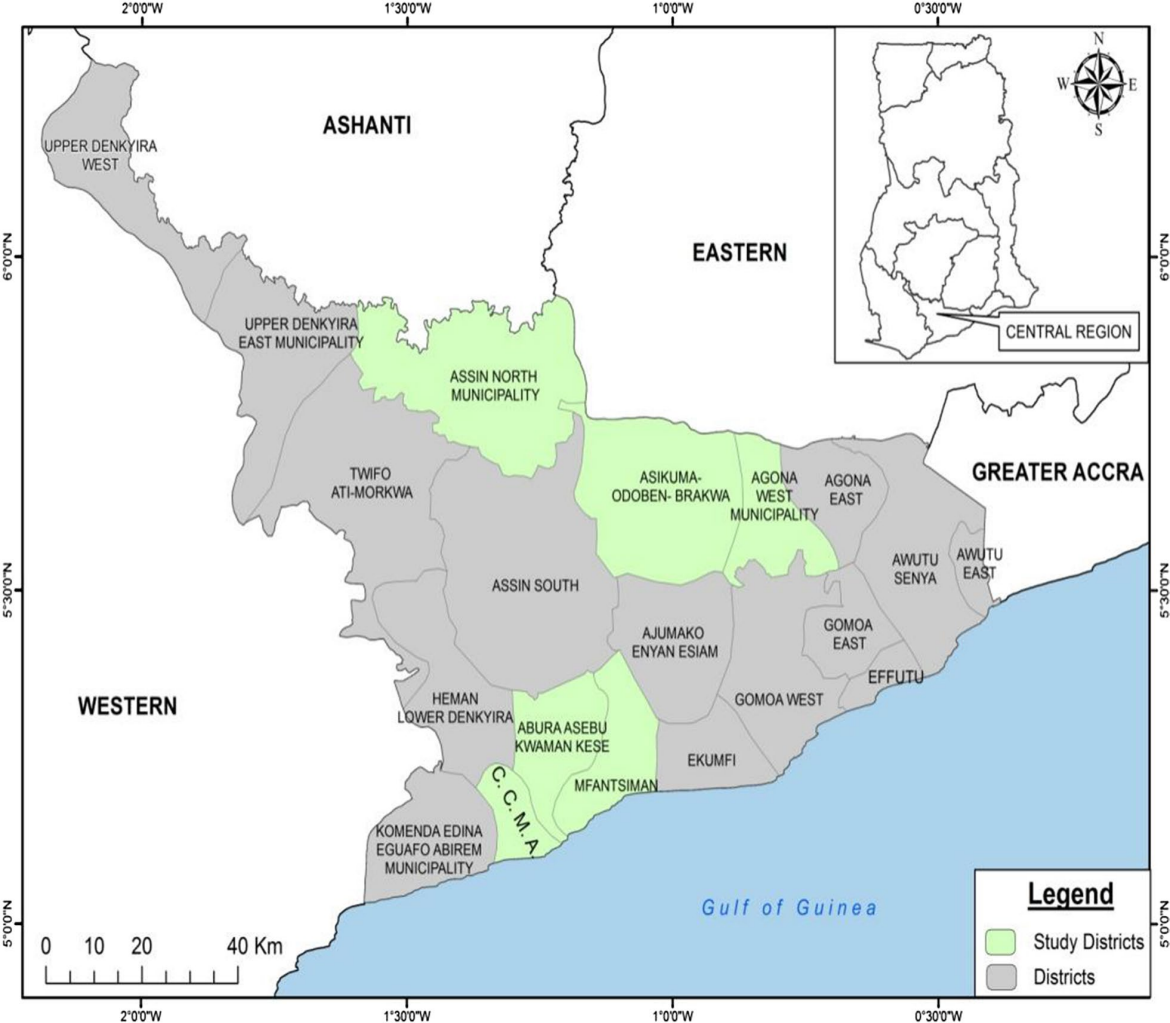
Map of the study area. *Source:* Department of Geography of the University of Cape Coast [23]

The population comprised women with HIV that received treatment at antiretroviral centres in the Central Region of Ghana as well as the general population of women in the Region that received care at the Outpatient Departments (OPDs) in the selected health facilities. The population of women with HIV in the Central Region was estimated to be 6019 [22]. Women who attended the hospital for general outpatient department services other than HIV care constituted the population of those with unknown HIV status. HIV status in this study was verified by including women known to be living with HIV receiving care for HIV at the varied Antiretroviral centres during their clinic days. The women were involved after they had been seen by a doctor or HIV nurse prescriber at the clinic. The Central Region is one of the 16 regions in Ghana located in the southern part of the country. The Region has health facilities at all levels of the health care delivery and most of the facilities have been equipped to provide HIV care. Figure 1 shows the districts in the Central Region where the data was collected.

### Sample and sampling

Based on Ogah [24] formula for non-experimental designs, a sample size of 600 was arrived at for both women diagnosed with HIV and those with unknown HIV status.

$${\text{Sample\;size}} = \surd {\text{n}} = \surd {\text{p}}\;(1 - {\text{p}}) \times {\text{cl}}/{\text{ci}}$$

√n = square root of sample size, *p* = variability or probability = 0.5 cl = confidence level = 95%. ci = confidence interval = ± 4%.

$$\begin{aligned} {\text{Sample\;size}} & = 600.25 \\ & \cong 600 \\ \end{aligned}$$.

Six health facilities involved in HIV care in the Central Region of Ghana were randomly sampled for the study. The probability proportion to size method was used in determining the participants to be included from each health facility. Convenience sampling method was used by including women with HIV who visited the selected health facilities and were willing to participate in the study. In the same way, women who visited the facility for general OPD services were approached and those who volunteered to participate in the study were included. For a uniform comparative study, equal sub-samples were selected for participants with and without HIV from each health facility. Eventually, 1200 participants comprising 600 participants with HIV and another 600 without HIV were used for the study.

### Data collection

A structured interview schedule was adopted for this study [15]. The instrument focused on perceived susceptibility, perceived benefits and sociodemographic factors. The items on the perception of susceptibility to cervical cancer and benefits of cervical cancer screening subscales were measured on a four-point Likert scale representing their level of agreement or disagreement, ranging from strongly agree, agree, disagree to strongly disagree. The respondents indicated their level of agreement or disagreement by responding to the questions on the subscales. The sociodemographic information of the respondents included age, marital status, religion, level of education, and employment status. The instrument was validated in the current study with a Cronbach’s alpha internal consistency indices of 0.826 for the perceived susceptibility subscale and 0.799 for the perceived benefits subscale.

Participants were recruited from varied health facilities. Six professional nurses were trained as research assistants. The training covered how they should ask the questions as well as things they should avoid during the data collection process thereby limiting the introduction of errors into the data. The structured interview schedule was administered face to face. The data were collected at the ART centres for women with HIV and OPDs for those with unknown HIV status. The interviews were conducted in the local dialect, Fante, and few conducted in English. The interviewers were fluent in both languages and they had experience of working with people living with HIV in the study sites. The data were collected between March and May 2016. The data collection for those with HIV took place at the counseling rooms in the respective health facilities, while that for women with unknown HIV status were taken either before or after being taken care of at a private place at the OPD.

### Data analysis

The data were analysed using the Statistical Package for Social Sciences software version 21.0 (IBM Corporation, Armonk, NY, USA). Statistical tests used were frequency counts, percentages, chi-square and independent sample t-test. The four-point Likert scale type questions were dichotomised into either agree or disagree at the point of the analysis to determine the association between perceived susceptibility to cervical cancer, perceived benefits of cervical cancer screening and HIV status. The t-test was used to compare the perceptions of susceptibility to cervical cancer and benefits of cervical cancer screening among women diagnosed with HIV and those with unknown HIV status.

## Results

Table 1 presents the association between sociodemographic characteristics and HIV status of the respondents. It shows that 26.3% (n = 158) of the women diagnosed with HIV were between the age range 30–39 years while 25.0% (n = 150) of those with unknown HIV status were within that age range. Additionally, 21.2% (n = 127) of those diagnosed were within the age range 20–29 years. Regarding the marital status of the respondents, 40.3% (n = 242) of those living with HIV were married, 23.3% (n = 140) were widowed while 13.5% (n = 81) were cohabiting. Compared to those with unknown HIV status, 58.7% (n = 352) were married, 7.5% (n = 45) were widowed and 1.8% (n = 11) were cohabiting. Again, 40.8% (n = 245) of those diagnosed and 20.3% (n = 122) of those with unknown HIV status had secondary education, respectively. More than half of those with HIV, 55.0% (n = 330), were students while 4.0% (n = 24) of those with unknown HIV status were students. Again, 39.7% (n = 238) of those diagnosed were unemployed compared to 13.0% (n = 78) of those with unknown HIV status. Table 1 further shows a statistically significant association between marital status (X^2^ = 167.071, *p* = 0.001), religion (X^2^ = 57.720, *p* = 0.001), level of education (X^2^ = 118.997, *p* = 0.001) and employment status (X^2^ = 782.646, *p* = 0.001), and HIV status. However, age did not have significant association with HIV status (X^2^ = 3.769, *p* = 0.001).

**Table 1:** Association between sociodemographic factors and HIV Status N = 1200

Sociodemographic factors	HIV status unknown f (%)	Diagnosed HIV status f (%)	X^2^	*p* value
*Age range*				
20–29	135 (22.5)	127 (21.2)		
30–39	150 (25.0)	158 (26.3)		
40–49	152 (25.3)	148 (24.7)		
50–59	130 (21.7)	119 (19.8)		
Above 59	33 (5.5)	48 (8.0)	3. 769	*p* = 0.001
*Marital status*				
Single	123 (22.8)	52 (8.7)		
Married	352 (58.7)	242 (40.3)		
Divorced	55 (9.2)	85 (14.2)		
Widowed	45 (7.5)	140 (23.3)		
Cohabiting	11 (1.8)	81 (13.5)	167.071	*p* = 0.001
*Religion*				
Christianity	597 (99.5)	537 (89.5)		
Islam	3 (0.5)	63 (10.5)	57.720	*p* = 0.001
*Level of education*				
No formal education	123 (20.5)	127 (21.2)		
Primary	293 (48.8)	125 (20.8)		
Secondary	122 (20.3)	245 (40.8)		
Tertiary	62 (10.3)	103 (17.2)	118.997	*p* = 0.001
*Employment status*				
Retired	6 (1.0)	12 (2.0)		
Student	24 (4.0)	330 (55.0)		
Unemployed	78 (13.0)	238 (39.7)		
Employed	492 (82.0)	20 (3.3)	782.646	*p* = 0.001

f denotes frequency

Table 2 presents the association between perceived susceptibility to cervical cancer and HIV status. From Table 2 all the perceived susceptibility factors were statistically significant in associating with HIV status. For instance, “I had a relative with cancer, so I may get cervical cancer” (X^2^ = 14.083, *p* = 0.001), “I have been in a polygamous relationship, so I may get cervical cancer” (X^2^ = 106.979, *p* = 0.001), “I may not get cervical cancer because I hardly have sex” (X^2^ = 74.949, *p* = 0.001) and “I do not feel at risk of getting cervical cancer” (X^2^ = 62.403, *p* = 0.001) have all significantly associated with HIV status.

**Table 2:** Association between perceived susceptibility to cervical cancer and HIV status of women

Perceived susceptibility factors	Unknown HIV status n (%)	Diagnosed HIV status n (%)	X^2^	*p* value
*I worry about developing cervical cancer*				
Agree	250 (41.7)	186 (31.0)		
Disagree	350 (58.3)	414 (69.0)	28.203	*p* = 0.001
*I had a relative with cancer, so I may get cervical cancer*				
Agree	107 (17.8)	105 (17.5)		
Disagree	493 (82.2)	495 (82.5)	14.083	*p* = 0.001
*I don’t think I can get cervical cancer*				
Agree	339 (56.5)	389 (64.8)		
Disagree	261 (43.5)	211 (35.2)	51.220	*p* = 0.001
I have been in a polygamous relationship so, I may get cervical cancer				
Agree	243 (40.5)	348 (58.0)		
Disagree	357 (59.5)	252 (42.0)	106.979	*p* = 0.001
*I may not get cervical cancer because I hardly have sex*				
Agree	343 (57.2)	342 (57.0)		
Disagree	257 (42.8)	258 (43.0)	74.949	*p* = 0.001
*I think I may get cervical cancer sometime in my life*				
Agree	225 (37.5)	227 (37.8)		
Disagree	375 (62.5)	373 (62.2)	53.149	*p* = 0.001
*I don’t feel at risk of getting cervical cancer*				
Agree	350 (58.3)	374 (62.3)		
Disagree	250 (41.7)	226 (37.7)	62.403	*p* = 0.001

Specifically, compared to women with unknown HIV status, 58.0% (n = 348) of those diagnosed with HIV agreed that they have been in polygamous relationships so they may get cervical cancer. Compared to 42.8% (n = 257) of those with unknown HIV status who disagreed that they may not get cervical cancer because they hardly have sex, 43.0% (n = 258) of those diagnosed with HIV disagreed to this same statement. Regarding the statement “I don’t feel at risk of getting cervical cancer”, 41.7% (n = 250) of the women with unknown HIV status disagreed to this statement while 37.7% (n = 226) of the women diagnosed also disagreed to the statement. However, 62.3% (n = 374) of the women diagnosed agreed to the statement. The majority of those diagnosed 82.5% (n = 495) disagreed to the statement that they had a relative with cancer so they may get cervical cancer.

Table 3 presents the association between perceived benefits of cervical cancer screening and HIV status. From Table 3, all the perceived benefits factors were significantly associated with HIV status. For instance, “screening can find cervical changes” (X^2^ = 7.120, *p* = 0.001), “screening can’t prevent the spread of cervical cancer” (X^2^ = 105.628, *p* = 0.001) and “screening can’t save the patient life” (X^2^ = 80.500, *p* = 0.001) had statistically significant associations with HIV status. Specifically, a high proportion of women diagnosed 94.8% (n = 569) and those with unknown HIV status 93.5% (n = 561) agreed that “screening can find cervical changes”. Also, 26.0% (n = 156) of those diagnosed compared to 29.5% (n = 177) of those with unknown HIV status agreed that “cervical screening may cause infertility”.

**Table 3:** Association between perceived benefits of cervical cancer screening and HIV status

Perceived benefits factors	Unknown HIV status n (%)	Diagnosed HIV status n (%)	X^2^	*p* value
*Screening can find cervical changes*				
Agree	561 (93.5)	569 (94.8)		
Disagree	39 (6.5)	31 (5.2)	7. 120	*p* = 0.001
*If cervical changes are found early, they are easily treated*				
Agree	569 (94.8)	586 (97.7)		
Disagree	31 (5.2)	14 (2.3)	9.252	*p* = 0.001
*Screening will help a woman to know if she has cancer*				
Agree	563(93.8)	587 (97.9)		
Disagree	37 (6.2)	13 (2.1)	16.806	*p* = 0.001
*Screening can’t prevent the spread of cervical cancer*				
Agree	293 (48.8)	135 (22.5)		
Disagree	307 (51.2)	465 (77.5)	105.628	*p* = 0.001
*Screening can’t save the patient life*				
Agree	253 (42.2)	124 (20.7)		
Disagree	347 (57.8)	476 (79.3)	80.500	*p* = 0.001
*Cervical screening may cause infertility*				
Agree	177(29.5)	156 (26.0)		
Disagree	423 (70.5)	444 (74.0)	35.923	*p* = 0.001

Table 4 presents the results of the independent samples t-test performed on women diagnosed with HIV and those with unknown HIV status in relation to perceived benefits and perceived susceptibility to cervical cancer. A comparison of the mean difference for women diagnosed and those with unknown HIV status in relation to perceived benefits showed a statistically significant difference for perceived benefits of cervical cancer screening (t = 7.418, df = 1198, *p* = 0.001). Nonetheless, there was no statistically significant difference in the means for women diagnosed and those with unknown HIV status regarding perceived susceptibility to cervical cancer (t = 0.935, df = 1198, *p* = 0.351).

**Table 4:** Independent samples t-test on perceived susceptibility and benefits and HIV status

Variables	HIV status	N	Mean	SD	Mean Diff	df	t-value	*p* value
Susceptibility	Unknown	600	18.205	3.785	0.262	1198	0.932	0.351
	WLHIV	600	18.467	5.738				
Benefits	Unknown	600	20.415	2.675	1.493	1198	7.418	0.001*
	WLWHIV	600	21.908	4.143				

*Significant at 0.01 significance level

## Discussion

### Association between sociodemographic factors and HIV status

The findings suggest an association between marital status and HIV status. In this study, married women had a higher rate of not knowing their HIV status 58.7% compared to those who were single 22.8%. Also, 13.5% of women in cohabitation were diagnosed against 1.8% of those with unknown HIV status. A possible explanation could be that women in marital relationships may have low perception of susceptibility to HIV, especially if the relationship is stable and monogamous in nature. The findings from a South African HIV prevalence survey posit that married women living with their spouses had reduced chances of being diagnosed HIV-positive compared to all other marital status categories, with the highest prevalence of HIV infection being recorded in those in cohabitation [25, 26]. In the present study, 13.5% of those diagnosed with HIV were in cohabitation. This makes cohabitation an important risk factor for HIV infection. Earlier studies conducted in South Africa and Zambia affirmed that women who were single and widowed had higher risk of HIV infection compared to those who were married [27, 28]. Women in such unstable relationships may have low social interconnections and socioeconomic status which can possibly explain their high risk for HIV [25]. The association between marital status and HIV is multifaceted demanding context specific approaches. Consequently, interventions for HIV prevention needs to target women in unstable marital relationships [25] in order to achieve maximum impact.

Religion was statistically significant in associating with HIV status. Among the women with unknown HIV status, 99.5% were Christians against 0.5% being Muslims. Similarly, 89.5% of the women diagnosed were Christians while 10.5% of them were Muslims. This finding is not surprising as the study was conducted in the southern part of Ghana which is a Christian dominated area and that may have accounted for the differences observed in the proportions. Consistent with this finding is a cross-sectional study conducted among 15-to-24-year-olds in Uganda which reported higher incidence of HIV among Christians compared to Muslims [29] suggesting the need for faith-based methods in HIV prevention. In a Malawian study, men in the Pentecostal churches were found to have decreased perceived risk regarding HIV risk behaviour. This supports the assertion that active participation in religious programmes may enable men to eliminate untoward behaviours such as extramarital partners [30].

In this study, level of education statistically significantly associated with HIV status. It was realized that among women with no formal education, there was no significant difference between those with unknown HIV status and those diagnosed. Those with unknown HIV status represented 20.5% of the total number while those diagnosed represented 21.2% of the total number. Nonetheless, among those diagnosed, 40.8% had secondary education compared to 20.3% of those with unknown HIV status. Most of the women with unknown HIV status had primary education. A likely explanation for the increase in HIV infections among those with secondary education is the evidently high-risk sexual behaviours among this population in the Ghanaian context [31]. Furthermore, 55.0% of those diagnosed with HIV in this study were students. A previous study conducted in Nigeria found the highest prevalence of HIV infection among women with secondary level of education [32]. The findings of a previous study indicate that women with less education were reported to have more HIV infections compared to those with tertiary education [27]. An earlier study conducted in some eastern and southern African countries suggests that those out of school had higher odds of being HIV-positive [33]. Contrary, findings from the Demographic and Health Survey data of nine countries in SSA region showed that being in school was associated with decreased likelihood of being infected with HIV in Uganda, Lesotho and Swaziland but there was no statistically significant evidence in Tanzania, Kenya, Zambia, Mozambique, Malawi and Zimbabwe. In relation to the current study, the context specific issues could have accounted for the differences in findings [33].

Comparing employment status with HIV status, it was statistically indicated that 55.0% of the women diagnosed were students followed by those who were unemployed 39.7%. This means that the socioeconomic backgrounds of women could possibly explain the risk for HIV infection. A population-based study conducted among teenagers in Zimbabwe found a high risk of HIV infection among individuals who were out of school and those unemployed [28]. The heterogeneity in the population characteristics could explain the observed findings. Evidence from Uganda suggest that being infected with HIV reduced the likelihood of being employed among males. It is worth noting that having a CD4 cell counts above 200 per mm^3^ increases the chances of being employed compared to those with low CD4 cell counts [34]. An earlier cross-sectional survey further highlighted that those women with HIV not working were less likely to accept that work was essential for their physical and mental wellbeing but were of the view that they needed to work only when they feel absolutely well compared to the working class [35]. Nonetheless, a high proportion of those with unknown HIV status (82.0%) were employed with only 4.0% being students.

However, age was not statistically significantly associated with HIV status, but the descriptives suggest that women who fell within the range of 20–29 years were about 1 out of 5 for those with unknown HIV status (22.5%) and (21.2%) for those diagnosed with HIV. Moreover, those within the age range of 30–39 formed about a quarter each of the participants for unknown HIV status (25.1%) and diagnosed HIV status (26.3%), respectively. Similarly, a quarter each of those with unknown HIV status (25.3%) and diagnosed were within the age range of 40–49 years. Therefore, there were no difference in HIV status in relation to these age categories for women diagnosed and those with unknown HIV status. An earlier study conducted in South Africa indicates that older age and being a female was associated with HIV testing [36]. Females are more likely to discuss health promoting actions with guardians and other significant others.

### Association between perceived susceptibility to cervical cancer and HIV status

The findings show that all the perceived susceptibility factors were associated with HIV status. Compared to women with unknown HIV status 40.5% (n = 243), 58.0% (n = 348) of women with unknown HIV status agreed that they have been in polygamous relationships so they may get cervical cancer. The nature of this kind of relationship put women at high risk for sexually transmitted infections. Multiple sex partners, polygamy, and exposure to sexually transmitted infections have been reported as important risk factors for cervical cancer [37, 38]. Earlier studies reported positive correlation between the number of sexual partners and perceived susceptibility to cervical cancer [39, 40]. Moreover, 42.8% (n = 257) of the women with unknown HIV status disagreed that they may not get cervical cancer because they hardly have sex while 43.0% (n = 258) of those diagnosed with HIV disagreed to the same statement. It is plausible to assume that these women knew that sexual intercourse was a major medium for HPV infection and exposure does not depend on the frequency of sexual intercourse, but unprotected sex with an exposed individual can lead to the disease.

Regarding the statement “I don’t feel at risk of getting cervical cancer”, 41.7% (n = 250) of women with unknown HIV status disagreed while 37.7% (n = 226) of those diagnosed disagreed. Nonetheless, 62.3% (n = 374) of those diagnosed agreed to the statement. This means a significant proportion of women diagnosed with HIV did not feel at risk of getting cervical cancer. This suggests the need for interventions that will facilitate WLHIV to evaluate their level of risk regarding cervical cancer. It is interesting to note that there was not much variation in the proportion of women with unknown HIV status and those diagnosed with HIV in relation to the statement “I think I may get cervical cancer sometime in my life”. In a previous study, perception of risk of cervical cancer was found to be low in women who considered themselves at low risk and had information about the risk factors [41]. Empirical evidence by Garces-Palacio and Scarinci [42] and Gu et al. [11] suggests that the majority of women viewed themselves to be at low risk of developing cancer of the cervix. Health education on the risk factors may decease risk perception regarding the disease [38]. Moreover, previous exposure to a sexually transmitted infection, level of education, having thoughts of being at risk of HPV, and having a family member or relative diagnosed with cervical cancer have been identified as risk factors. Perception of susceptibility was highly associated with a present or past perception of exposure to HPV [42]. The synergy between cervical cancer and HIV have been established, requiring women with HIV to take measures to prevent cervical cancer [3]. In Kenya, women with greater chances of being diagnosed with HIV in the past four years felt at risk of developing cervical cancer and were willing to seek screening services if available [9]. Additionally, the belief of being at risk increased the likelihood of going for cervical cancer screening [43].

### Association between perceived benefits of cervical cancer screening and HIV status

The findings further indicate an association between all the perceived benefits factors and HIV status. Specifically, a high proportion of women diagnosed with HIV 94.8% (n = 569) and those with unknown HIV status 93.5% (n = 561) agreed that “screening can find cervical changes”. The high perception of benefits observed in this study could be due to increased awareness about the disease through mass media campaigns. Despite the role of cervical cancer screening in the detection of cervical abnormalities early before developing into cancer, 42.2% of the women with unknown HIV status held the view that screening cannot save lives. This calls for enhanced sensitization among the general population to increase their knowledge and understanding about the disease and clear misconceptions people might have regarding cervical cancer screening. Interestingly, previous studies have highlighted the immense role of cervical cancer screening in saving the lives of women as it decreases the incidence, morbidity and mortality associated with the disease [17]. In a related study, the women viewed cervical screening to be necessary, as it could identify cervical changes early before it progresses into cancer [18]. The screening test might facilitate early treatment and possible cure due to early detection of cervical abnormalities [18, 19]. While the advantages of cervical cancer screening far outweigh the potential harm, a majority of women lacked information about the benefits of screening and wanted this vital information before participating in any cervical cancer screening tests [44]. The findings of a cross-sectional study conducted by Luszczynska, Durawa, Scholz and Knoll [45] indicate a significant relationship between perception of benefits and use of cervical screening facilities. Perceived benefits predicted intention to have cervical cancer screening in an earlier study [20]. Nonetheless, some misconceptions regarding the benefits of screening have been observed in the current study. For instance, 26.0% (n = 156) of women diagnosed compared to 29.5% (n = 177) of those with unknown HIV status agreed that “cervical screening may cause infertility”. Education on cervical cancer screening for both women diagnosed and those with unknown HIV status is critical in enabling them take informed decisions.

### Differences in perceived susceptibility to cervical cancer and perceived benefits of cervical cancer screening in relation to HIV status

The findings show a statistically significant difference between women diagnosed with HIV and those with unknown HIV status regarding the perceived benefits of cervical cancer screening. The result indicates that those diagnosed of HIV tend to perceive significantly higher benefits than women of unknown HIV status. A possible explanation could be that those diagnosed may be aware of their immunocompromised state and the possibility that they may get cervical cancer. They might have heard about the fact that screening is one of the most effective interventions in preventing cervical cancer. A previous study conducted among HIV-positive women in the Central Region of Ghana reported that women with HIV had higher benefits of cervical cancer screening and consequently had the intention of screening [15]. Similarly, an intervention study conducted in the Komenda, Edina, Eguafo, Abirem district of Ghana found out that women in the intervention viewed cervical cancer screening to be more beneficial compared to the control group [46]. This suggests the need for increased education about screening, even among women in general and not necessary the high-risk group since all sexually active women are at risk of cervical cancer. Moreover, a community-based study conducted in Ethiopia reported high benefits among the general women studied [47]. Perhaps there has been more sensitization programmes about cervical cancer and screening modalities in that context.

Furthermore, the findings show no significant difference in the level of perceived susceptibility to cervical cancer between women of unknown HIV status and those diagnosed in relation to their HIV status. This indicates that both groups may or may not feel susceptible to cervical cancer which requires intensive education about the disease. Women with HIV have higher tendency of developing cervical cancer as the disease progresses faster in people with weak immune system. Also, the association between immunosuppression, HPV infection and cervical cancer has long been established [3, 48]. The level of susceptibility of women with HIV regarding cervical cancer requires further exploration, as interventions to increase screening uptake may be successful if they feel at risk of the disease. In an earlier health education intervention study conducted among women with unknown HIV status in Ghana, perceived susceptibility decreased after the intervention compared to the control group [46]. This indicates that regardless of the HIV status women generally have poor susceptibility perception towards cervical cancer. Consequently, other novel methods of educating the women need to be explored and implemented to enable them appreciate their level of risk for cervical cancer and adopt measures to prevent it.

## Conclusions

Women with HIV had higher perceived benefits of cervical cancer screening compared to those with unknown HIV status which is a positive outcome in efforts to decrease the incidence of cervical cancer in high-risk populations. However, there was not any significant difference regarding perceived susceptibility for those diagnosed with HIV and those unknown to have the disease. This finding calls for interventions to increase awareness on cervical cancer, especially the risk factors to enable women understand and evaluate their level of susceptibility to the disease. Moreover, interventions for women with HIV should consider some important sociodemographic factors.

## Data Availability

The datasets used and/or analysed during the current study are available from the corresponding author on reasonable request.

## Abbreviations

AIDS: Acquired Immunodeficiency syndrome
ART: Anti-retroviral therapy
CD4: Cluster of differentiation 4
HPV: Human papillomavirus
HIV: Human immunodeficiency virus
OPD: Out-patient department
PLHIV: People living with HIV
SSA: Sub-Saharan Africa
UNAIDS: The Joint United Nations Programme on HIV/AIDS
WHO: World Health Organisation
WLHIV: Women Living with HIV
